# Potential Mechanisms of Change in Virtual Reality-based Cognitive Behavioural Therapy and Standard Cognitive Behavioural Therapy for Schizophrenia-Related Paranoia: A Mediation Analysis from the FaceYourFears Trial

**DOI:** 10.1093/schizbullopen/sgag028

**Published:** 2026-07-21

**Authors:** Ulrik N Jeppesen, Ditte L Vernal, Mads J Christensen, Amy E Pinkham, Wim Veling, Marit Hidding, Sara N Jensen, Stephen F Austin, Merete Nordentoft, Louise B Glenthøj

**Affiliations:** VIRTU Research Group, Mental Health Center Copenhagen, Copenhagen University Hospital, Mental Health Services CPH, 2900 Hellerup, Capital Region of Denmark, Denmark; Department of Psychology, University of Copenhagen, 1353 Copenhagen, Capital Region of Denmark, Denmark; Department of Psychiatry, Aalborg University Hospital, 9260 Gistrup, North Denmark Region, Denmark; Department of Psychiatry, Aalborg University Hospital, 9260 Gistrup, North Denmark Region, Denmark; Department of Clinical Medicine, Faculty of Medicine, Aalborg University, 9260 Gistrup, North Denmark Region, Denmark; Department of Psychiatry, Aalborg University Hospital, 9260 Gistrup, North Denmark Region, Denmark; Department of Clinical Medicine, Faculty of Medicine, Aalborg University, 9260 Gistrup, North Denmark Region, Denmark; School of Behavioral and Brain Sciences, University of Texas at Dallas, Richardson, TX 75080, United States; University of Groningen, Faculty of Medical Sciences, University Medical Center Groningen, 9713 GZ Groningen, The Netherlands; University of Groningen, Faculty of Medical Sciences, University Medical Center Groningen, 9713 GZ Groningen, The Netherlands; Sanos, Copenhagen, 2860 Søborg, Capital Region of Denmark, Denmark; Department of Psychology, University of Copenhagen, 1353 Copenhagen, Capital Region of Denmark, Denmark; Mental Health Services East, Copenhagen University Hospital—Psychiatry, 4000 Roskilde, Region Zealand, Denmark; VIRTU Research Group, Mental Health Center Copenhagen, Copenhagen University Hospital, Mental Health Services CPH, 2900 Hellerup, Capital Region of Denmark, Denmark; Department of Clinical Medicine, Faculty of Health and Medical Sciences, University of Copenhagen, 2200 Copenhagen, Capital Region of Denmark, Denmark; VIRTU Research Group, Mental Health Center Copenhagen, Copenhagen University Hospital, Mental Health Services CPH, 2900 Hellerup, Capital Region of Denmark, Denmark; Department of Psychology, University of Copenhagen, 1353 Copenhagen, Capital Region of Denmark, Denmark

**Keywords:** RCT, psychotic disorders, psychotherapy, CBT, VR

## Abstract

**Background and Hypothesis:**

Cognitive models conceptualize paranoia in schizophrenia spectrum disorders as driven and maintained by mechanisms, which can be targeted by cognitive behavioral therapy for paranoia (CBTp). This study examined potential mechanisms as mediators. Safety behaviors, belief inflexibility, and core beliefs about self and others were hypothesized to differentially mediate outcomes in virtual reality-based CBTp (VR**–**CBTp) and CBTp, with safety behaviors expected to be more influential in VR**–**CBTp, and belief inflexibility and self-related core beliefs in CBTp.

**Study Design:**

This secondary analysis of the FaceYourFears trial included a per-protocol sample with complete data (VR**–**CBTp: *n =* 96; CBTp: *n =* 90). Groups were comparable, except for greater in-session exposure in VR**–**CBTp. Pearson correlations, linear regression analyses and structural equation modeling (SEM), examined each mediator separately. Causal inferences could not be drawn.

**Study Results:**

Pearson correlations of change scores showed that positive safety behaviors and negative self- and others-related core beliefs were associated with paranoia across both interventions. Regression analyses without covariate adjustment showed no significant between-group differences, except for belief inflexibility (*P* = .041), which became non-significant with covariate adjustment (age, sex, depression). SEM showed no differential mediation effects.

**Conclusions:**

No mediators differed between interventions, and no hypotheses were supported. These results suggest similar mechanistic pathways in CBTp and VR**–**CBTp. Treatment selection may instead best be guided by factors such as symptom profile, patient preference, accessibility, and engagement. Findings are discussed in relation to prior mediation studies within the field, with implications for future research.

## Introduction

Paranoia in schizophrenia spectrum disorders (SSD) is in this study defined as ideas of persecution and social self-reference, spanning a continuum from transient suspiciousness to fully formed delusion.^1^ This definition is situated within cognitive models that conceptualize paranoia as a universal human experience.^2–7^

Cognitive behavioral therapy for paranoia (CBTp), grounded in cognitive models, has become a central psychotherapeutic approach for treating paranoia in SSD over recent decades.^4^ An umbrella review supports the efficacy of CBT for delusions, most commonly targeting paranoia, showing small to moderate effects at treatment completion.^8^ Broader CBT for psychosis approaches are also recommended in clinical guidelines.^9–11^

A key principle in cognitive models is the role of safety behaviors in maintaining paranoia.^4^^,^^7^ Safety behaviors are maladaptive strategies that provide short-term relief while maintaining the individual’s unreasonable belief by preventing disconfirmation. For instance, by avoiding eye contact in paranoia-inducing situations, the belief that others constantly display hostile looks cannot be challenged. Similarly, by avoiding previously paranoia-inducing situations, the belief that the situation is threatening cannot be disconfirmed. Exposure is considered a central treatment element for reducing safety behaviors,^4^ although its use is limited by practical barriers in clinical settings.^12^

To overcome this, virtual reality (VR) has been utilized for delivering exposure in an accessible and controlled format.^13^^,^^14^ In the FaceYourFears trial, we compared therapist-guided VR**–**CBTp with standard CBTp.^15^ As expected, VR**–**CBTp participants received significantly more in-session exposure than CBTp participants. However, as both interventions led to substantial and similar within-group reductions in paranoia, no significant between-group differences were observed at treatment cessation or at 6-month follow-up.

A plausible explanation for these findings is that other mechanisms of change may have been more strongly activated in standard CBTp, resulting in comparable paranoia reductions.

Psychotherapeutic trials using a “causal interventionist approach”^16^ have targeted different mechanisms, with their contribution varying according to therapy structure and delivery.^14^^,^^17–20^ The two interventions in FaceYourFears are theorized to target safety behaviors, reasoning biases (in the form of belief inflexibility), and core beliefs about self and others, albeit with different emphasis on each mechanism. These mechanisms, among others, are understood in models by Garety, Freeman, and colleagues as maintaining paranoia.^4^^,^^6^^,^^7^ Such models propose that, once formed, delusions are stabilized by maintaining mechanisms, and that targeting these through CBTp may weaken delusional conviction and distress.

Safety behaviors are linked to paranoia,^7^ and two VR**–**CBTp trials have identified them as mediators when explicitly targeted in exposure-based interventions.^14^^,^^20^ This is also reflected in our VR**–**CBTp treatment manual,^21^ where greater emphasis is placed on exposure to target safety behaviors than in standard CBTp.

Belief inflexibility is a reasoning bias implicated in both the development and maintenance of paranoia.^6^ Although defined somewhat differently across studies,^22–24^ a common feature is the tendency to disregard alternative explanations and to fail to acknowledge the possibility of being mistaken, resulting in more strongly held paranoid beliefs.^25^ This bias has been assessed using either delusion-specific^23^ or delusion-neutral content,^22^ depending on the measurement instrument. When assessed using delusion-specific content, belief inflexibility has been associated with paranoia^26^ and shown to mediate reductions in paranoia in non-VR trials^19^^,^^24^ that explicitly targeted this bias. In addition to CBT-based techniques, metacognitive strategies, originally developed by Moritz, Woodward, and colleagues,^27^ are used to target belief inflexibility. In our trial, the standard CBTp manual places greater emphasis on targeting belief inflexibility through cognitive restructuring and metacognitive strategies than VR–CBTp.

Core beliefs are cognitive generalizations shaped by past experiences that guide the interpretation of future situations.^28^ When maladaptive, such beliefs may bias the interpretation of social situations, for instance by fostering perceptions of threat and suspiciousness toward others.^29^ Core beliefs may therefore play a role in the emergence and persistence of paranoia. Paranoia has been shown to be linked to more negative and fewer positive self-related core beliefs, whereas evidence concerning others-related core beliefs remains inconsistent.^30–32^ Some trials suggest that self-related core beliefs may mediate treatment outcomes.^17^^,^^33^ In our trial, the CBTp manual places greater emphasis on enhancing self-confidence and thereby modifying self-related core beliefs than VR–CBTp.

By investigating these potential mechanisms of change, examined in this study as mediators, and how their roles may differ between interventions, this study aims to establish whether the two therapies benefit different patient subgroups and thereby inform treatment personalisation.^34^

Accordingly, we investigate (1) whether the potential mechanisms of change show indications of acting as mediators across groups, assessed through Pearson correlations of change scores, and in particular (2) whether their roles differ between VR–CBTp and CBTp, examined through linear regression models and structural equation modeling (SEM), with each mediator analysed separately.

Based on prior research and our treatment manuals, we hypothesize:

- Changes in safety behaviors will more strongly influence paranoia reduction in VR–CBTp compared to standard CBTp.
- Changes in belief inflexibility will more strongly influence paranoia reduction in standard CBTp compared to VR–CBTp.
- Changes in self-related core beliefs will more strongly influence paranoia reduction in standard CBTp compared to VR–CBTp.

The role of others-related core beliefs remains unclear and will be explored.

## Methods

### Participants and Procedure

The present study constitutes a secondary analysis of the FaceYourFears trial, a two-site, assessor-masked, randomized superiority trial conducted in the Capital Region of Denmark and the North Denmark Region between 26 March 2021 and 10 August 2024. The study protocol and its update were published in *Trials*,^15^^,^^35^ the main results were published in *Nature Medicine*,^36^ and a secondary moderator analysis has been published in *Psychological Medicine*.^37^ The trial investigated the efficacy of VR–CBTp compared to standard CBTp, both delivered over 10 sessions. Assessments were conducted at baseline, treatment cessation, and 6-month follow-up. No mid-treatment assessment of mediators or paranoia measures was collected, precluding the establishment of temporal order. Accordingly, mediation analyses can demonstrate associations but not causality.

Participants were recruited from public outpatient psychiatric services, primarily Flexible Assertive Community Treatment (F-ACT) teams and the OPUS program,^38^ a national early intervention service. Inclusion criteria were age ≥18, a diagnosis within the schizophrenia spectrum (ICD-10, F20-F29), providing informed consent, and at least mild paranoia operationalized as a Green Paranoid Thought Scale (GPTS) total score of ≥40. Exclusion criteria were known organic brain disease, estimated IQ ≤70, inability to tolerate assessments, or insufficient Danish language proficiency. All participants provided written informed consent. The final sample comprised 254 participants. The trial was approved by the Capital Region of Denmark Committee on Health Research Ethics (H-20048806) and the Danish Data Protection Agency (P-2020-823).

### Interventions

The treatment manuals were Danish adaptations of the VR–CBTp and CBTp manuals originally developed by the research team behind the Pot-Kolder et al., 2018 VR–CBTp trial,^14^ condensed from 16 to 10 sessions while preserving all core elements. Additionally, the manuals were revised based on feedback from individuals with lived experience. A treatment course of 10 individual sessions aligns with previous research^13^^,^^14^ and is consistent with symptom-specific and digital-enhanced guidelines.^39^

Both interventions were delivered by the same group of therapists, whose experience in CBT for psychosis ranged from 1 to 17 years. All underwent a 2-day training workshop covering both manuals. Two therapists, who joined the trial later, received individualized, side-by-side training from experienced colleagues. Ongoing internal group consultation and external expert supervision were provided throughout the trial period. Both VR–CBTp and standard CBTp adhered to a case formulation-based approach to treat paranoia and they were based upon cognitive models for paranoia.^4^^,^^6^^,^^7^^,^^40^^,^^41^ Fidelity to the treatment manuals was rated as “good” to “very good” in both interventions using an adapted version of the Cognitive Therapy Rating Scale^42^ that included intervention-specific components. Further, both durations, measured by therapists, and perceived quality, rated by participant’s, of in-session exposure were recorded in both interventions. A description of the interventions is provided as text in Supplementary Data Content 1.

### Measures

#### Paranoia

Level of paranoia was assessed using the Revised GPTS (R-GPTS)^43^; a self-report measure comprising two subscales: part A (8 items) assesses ideas of social self-reference, and part B (10 items) assesses ideas of persecution. Items are rated on a 5-point Likert scale (0-4), yielding a total score ranging from 0 to 72, with higher scores indicating greater symptom severity. The R-GPTS has excellent psychometric properties, including improved factor structure and internal consistency compared to the original GPTS, and was therefore selected as the outcome measure for paranoia in this secondary analysis.^43^

#### Safety Behaviors

Safety behaviors were assessed using the Safety Behavior Questionnaire (SBQ), a semi-structured interview with strong inter-rater and test–retest reliability.^44^ Each behavior was rated from 1 to 4 based on past-month frequency. Following recommendations, behaviors were categorized as negative (i.e. avoidance) or positive (active coping such as hypervigilance).^7^ Based on feedback from individuals with lived experience and supporting literature,^7^^,^^44^ we supplemented the in-situation subscale with closed-ended items to capture otherwise unrecognized behaviors. The SBQ showed excellent inter-rater reliability in the FaceYourFears trial.^36^ In this study, the SBQ positive and SBQ negative subscales, each ranging from 0 with no upper limit, were used to assess safety behaviors, with higher scores indicating greater symptom severity; these were conceptualized as mechanisms of change and analysed as potential mediators.

#### Core Beliefs of Self and Others

Core beliefs of self and others were measured by the self-report questionnaire Brief Core Schema Scale (BCSS).^29^ BCSS has displayed good psychometric properties and evidence of construct validity.^29^ It comprises four subscales that measure positive and negative beliefs about self and others. Each subscale consists of 6 items that are rated on a 5-point Likert scale with scores ranging from 0 to 24, where higher scores indicate a greater presence of the assessed core belief. For this study, the four subscales were used to examine core beliefs of self and others, conceptualized as mechanisms of change and analysed as potential mediators.

#### Belief Inflexibility

Belief inflexibility were assessed using the Davos Assessment of Cognitive Biases Scale (DACOBS),^22^ a 42-item self-report questionnaire with seven subscales. Each subscale consists of 6 items rated on a 7-point Likert scale, with total scores ranging from 6 to 42, where higher scores indicate greater symptom severity. Four subscales assess cognitive biases (jumping to conclusions, belief inflexibility, attention for threat og external attribution), two assess cognitive problems (social cognitive, subjective cognitive), and one assesses safety behaviours. The scale has high internal consistency and satisfactory test–retest reliability, with five subscales successfully validated. For this study, the belief inflexibility subscale was used to examine belief inflexibility, conceptualized as a mechanism of change and analysed as a potential mediator.

#### Depression

Depression at baseline was assessed as a covariate using the structured interview, Calgary Depression Symptom Scale (CDSS).^45^ It comprises 9 items rated from 0 (absent) to 3 (severe), with total scores ranging from 0 to 27, where higher scores indicate greater symptom severity. For this secondary study, scores were dichotomized (≥8) to indicate clinical depression (specificity: 91%, sensitivity: 85%) rather than analysed continuously. The selected cut-off reflects the use of the CDSS as a standalone measure rather than a screening tool leading to further diagnostic clarification and it was chosen to optimize the balance between false positive and false negative cases. This minimized symptom-level overlap with paranoia (e.g. ideas of self-reference^46^), allowing clinical depression to be examined specifically as a covariate.

### Statistical Analyses

The current study investigated potential mechanisms of change embedded within the full therapeutic protocol. Accordingly, the analyses were conducted on the per-protocol (PP) sample, defined as participants who completed all 10 treatment sessions (VR–CBTp: *n =* 102; CBTp: *n =* 97). This approach was made to ensure that all included participants were fully exposed to the range of therapeutic elements hypothesized to activate mechanisms of change between baseline and treatment cessation. While the original randomization helps to balance measured and unmeasured baseline characteristics, the PP analysis may not fully eliminate confounding due to the exclusion of non-adherent participants and the resulting risk of selection bias. Participants included in the PP sample may differ systematically from those excluded (e.g. in motivation, symptom severity, or other unmeasured factors), potentially compromising comparability between groups. Hence, we compared the PP sample with complete data in the present study to the intention-to-treat (ITT) sample from the main study, reported elsewhere,^36^ with respect to baseline characteristics and efficacy outcomes, as well as potential group differences between completers and non-completers, to assess potential selection bias. In addition, analyses adjusted for selected covariates were conducted to partly mitigate residual confounding; however, this does not fully address potential selection bias.^47^ Statistical analyses were performed by a statistician using R version 4.4.2 (2024-10-31 *ucrt*) and RStudio version 2024.12.1+563. Descriptive statistics in Table 1 were performed using SPSS version 29.0.1.0 (171).

**Table 1:** Clinical and Sociodemographic Characteristics of the Per-Crotocol Sample with Complete Data at Baseline.

Variable	VR**–**CBTp	CBTp	Total
Age in years	28.0 (23.0-35.0)^a^, *n* = 96	26.0 (23.0-33.0)^a^, *n* = 90	27.0 (23.0-34.0)^a^, *n* = 186
Sex			
Female	48 (50.0%)	53 (58.9%)	101 (54.3%)
Male	48 (50.0%)	37 (41.1%)	85 (45.7%)
Education			
Primary (in Denmark up until age 14)	26 (27.1%)	26 (28.9%)	52 (28.0%)
Secondary	32 (33.3%)	41 (45.6%)	73 (39.2%)
Vocational education	16 (16.7%)	9 (10.0%)	25 (13.4%)
University degree	22 (22.9%)	12 (13.3%)	33 (18.3%)
Other^b^	0	2 (2.2%)	2 (1.1%)
Occupational status			
Employment (full-time or part-time)	12 (12.5%)	21 (24.4%)	34 (18.3%)
Student	13 (13.5%)	22 (24.4%)	35 (18.8%)
Unemployment	48 (50.0%)	31 (34.4%)	79 (42.5%)
Disability retirement (or retired)	18 (18.8%)	12 (13.3%)	30 (16.1%)
Housewife or husband	1 (1.0%)	0	1 (0.5%)
Other	4 (4.2%)	3 (3.3%)	7 (3.8%)
Diagnosis			
F20 Schizophrenia	75 (78.8%)	63 (70.1%)	138 (74.2%)
F21 Schizotypal disorder	13 (13.5%)	18 (20.0%)	31 (16.7%)
F22 Paranoid psychoses	6 (6.3%)	3 (3.3%)	9 (4.9%)
F25 Schizoaffective psychosis	2 (2.0%)	2 (2.2%)	4 (2.1%)
F29 Non-organic psychosis	0	4 (4.4%)	4 (2.2%)
Outpatient routine care setting			
Early intervention services (the national OPUS program)	44 (45.8%)	45 (50.0%)	89 (47.8%)
Flexible Assertive Community Treatment teams (F-ACT)	52 (54.2%)	42 (46.7%)	94 (50.5%)
Other	0	3 (3.3%)	3 (1.6%)
Receiving antipsychotic medication (answering yes)	82 (85.4%)	73 (81.1%)	155 (83.3%)
Olanzapine equivalent (mg/day)	12.3 (6.2-20.0)^a^, *n =* 96	10.1 (5.8-18.5)^a^, *n =* 90	11.7 (6.0-20.0)^a^, *n =* 186
Participation in additional group psychoeducation as part of TAU from baseline to treatment cessation	9 (9.4%)	8 (8.9%)	17 (9.1%)
Participation in additional group social skills training as part of TAU from baseline to treatment cessation	10 (10.4%)	11 (12.2%)	21 (11.3%)
Participation in additional individual psychological counseling as part of TAU from baseline to treatment cessation	4 (4.2%)	5 (5.6%)	9 (4.8%)
Participation in additional other psychosocial intervention^c^ as part of TAU from baseline to treatment cessation	19 (19.8%)	11 (12.2%)	30 (16.1%)
R-GPTS total	28.5 (25.9-31.2%) (13.2), *n =* 96	28.2 (25.7-31.4%) (13.8), *n =* 90	28.3 (26.4-30.3%) (13.4), *n =* 186
SAPS composite total	23.5 (21.0-26.1%) (12.7), *n =* 96	20.7 (18.4-23.1%) (11.2), *n =* 90	22.2 (20.5-23.9%) (12.0), *n =* 186
In-session exposure in minutes	98.0 (89.3-106.6%) (42.7), *n =* 96	9.0 (4.7-13.3%) (20.4), *n =* 90	54.9 (46.8-63.0%) (55.9), *n =* 186

Values are presented as n(n%), indicating the frequency and its corresponding percentage of the total and n(n-n%), (n) indicating the mean value and its corresponding 95% confidence and its standard deviation. *n =* indicates number of observations. ^a^:indicates the median and its 25th and 75th percentile, presented as n(n-n), in non-normal distributed samples. ^b^: Education: “Other” refers to the lack of completion of Primary Education. ^c^: “other psychosocial intervention” refers primarily to family intervention. TAU: treatment as usual. R-GPTS: Revised Green Paranoid Thought Scale. SAPS: Scale for the Assessment of Positive Symptoms.

To examine associations between paranoia and the mediators at baseline and treatment cessation across interventions, Pearson correlations coefficients with corresponding *P*-values were calculated.

To address the first research aim, namely whether changes in the proposed mediators were associated with changes in paranoia across groups, Pearson correlation coefficients with corresponding *P*-values between change scores from baseline to treatment cessation were computed.

**Figure 1:**
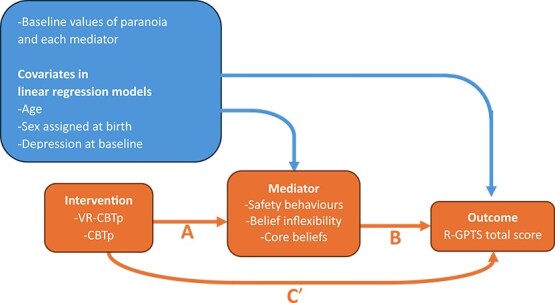
Causal Directed Acyclic Graph (Orange Boxes) Illustrating the Assumed Causal Model. Path A: Treatment Effect on Mediator; Path B: Mediator Effect on Outcome; Path C′: Direct Treatment Effect on Outcome. The Indirect (Mediated) Effect Is Estimated as A × B. Structural Equation Modeling Was Used to Test Group Differences in Direct and Indirect Effects for each Mediator. Blue Box: Linear Regression Models “without covariate adjustment” Were Adjusted for Baseline Values of Paranoia and each Mediator, and Further Baseline Covariates Were Included in the Linear Regression Models “with covariate adjustment” (Age, Sex Assigned at Birth, Depression at Baseline).

To address the second research aim, namely whether the associations between changes in the mediators and changes in paranoia differed between VR–CBTp and CBTp, separate linear regression models were fitted for each mediator. Each model included an interaction term between treatment and mediator, adjusted for baseline scores on both the R-GPTS and the mediator^48^ (Figure 1), to correctly capture the changes while accounting for the values at baseline. These models are consistently referred to as “without covariate adjustment” throughout. The estimate, standard error and corresponding two-sided *P*-value of the interaction term were then used to assess difference between the two treatment groups in the relationship between each mediator and R-GPTS. Residual plots and QQ-plots were tested for model diagnostics.

Separately for each mediator, an additional linear model was fitted adjusting for the prespecified covariates age, biological sex assigned at birth, and baseline depression (binary) (Figure 1). Throughout, these models are referred to as “with covariate adjustment”. Again, residual plots and QQ-plots were tested for model diagnostics. This model was fitted to account for potential confounding associated with the use of the PP sample and served as a sensitivity analysis.

For each mediator, linear model plots were generated using the *rms* package to display predicted values of the R-GPTS variable with 95% confidence intervals (95%CI). Predictions were shown across the full range of each mediator for both treatment groups, while holding continuous covariates at their median and categorical covariates at their reference level. These plots visually illustrate potential differences between treatment groups in the association between R-GPTS and the mediator under investigation.

To address the second research question further, SEM was used to test whether the mediators, explored separately, had different indirect (mediating) effects across the two treatment groups. SEM was conducted using the *sem* function from the *lavaan* package. Notably, measurement of the seven mediators and R-GPTS were conducted at the same time points, limiting the possibility of causal interpretation.^49^ Simple regression models were defined corresponding to a direct effect (C′) of treatment on R-GPTS, an effect (A) of treatment on mediator, and an effect (B) of mediator on R-GPTS (Figure 1). As a general guideline, 200 data points are typically required for SEM analyses. However, due to the simple structure of the model used in this study, where each mediator was analysed separately, the available 186 data points have been deemed sufficient for conducting SEM analyses. To ensure valid inference, 5000 bootstrap resamples were used to compute 95%CI (based on the percentiles of the bootstrap distribution). The estimates, SE, and 95%CI of the direct effect (C′), indirect effect (AB), and total effect (C′ + AB) are reported as well as the indirect effect divided with the total effect to show the proportion mediated.

No formal adjustments for multiplicity were applied, as the predefined hypotheses were tested using different types of analyses rather than a single family of tests. Further, the study was not powered for the mediation analyses, but to test the hypotheses in the main trial.^36^

### Post Hoc Analyses of Exposure

To further examine the role of exposure within each group, we conducted post hoc analyses. We assessed the association between in-session exposure and reduction in paranoia separately for each group using linear regression models. As in the primary analyses, models were estimated both with and without covariate adjustment.

## Results

### Participant Characteristics

The PP sample comprised of the 199 participants who received full treatment (i.e. 10 sessions), representing 78.3% of the 254 randomized participants. As reported elsewhere,^36^ the proportion of completers and non-completers did not differ significantly between groups (*P* = .09). Across all variables in the current study, 6.5% of the sample (*n =* 13) was excluded from the regression analyses due to incomplete data. The final analytic sample consisted of 186 participants, with 96 allocated to VR–CBTp and 90 to CBTp.

The final sample comprising of the PP sample with complete data were consistent with the baseline characteristics of the ITT sample, also reported elsewhere.^36^ In the ITT sample, participants across groups included a slightly higher proportion of females, had lower levels of education, and were less severely ill (see Table 1 and table, Supplementary Data Content 2).

Sociodemographic and clinical characteristics were overall well balanced between groups at baseline. The most notable difference was a small age difference, with the CBTp group being younger and consequently including more students and fewer participants with a completed education. As the difference in educational attainment appeared to be largely attributable to this age difference, it was not considered to warrant inclusion as an additional covariate beyond those prespecified in the statistical analysis plan. The only substantial difference was the expected difference in in-session exposure, which was substantially greater in the VR–CBTp group due to the treatment design (Table 1).

In addition, results from the main ITT analysis and PP sensitivity analyses examining changes in R-GPTS total and the seven mediators from baseline to treatment cessation, as reported elsewhere,^36^ are presented (see table, Supplementary Data Content 3 and 4). These analyses yielded consistent results across the two samples, with no between-group differences for any examined variables.

### Pearson Correlations Between Paranoia and Mediators

At baseline, all candidate mediators, except positive self-related core beliefs, were significantly associated with R-GPTS (see table, Supplementary Data Content 5). In particular, negative (r = 0.40) and positive (r = −0.33) other-related core beliefs were moderately correlated with R-GPTS. At treatment cessation, most correlations were further strengthened, except for positive others-related core beliefs (see table, Supplementary Data Content 5).

Regarding change between baseline and treatment cessation, most mediators were significantly associated with change in R-GPTS total score, apart from belief inflexibility and positive self-related core beliefs (see table, Supplementary Data Content 5). The largest statistically significant associations were observed for positive safety behaviors (r = 0.19, *P* < .01) and negative self- and other-related core beliefs (negative self: r = 0.19, *P* < .01; negative others: r = 0.21, *P* < .01), indicating small correlations (see table, Supplementary Data Content 5).

### Linear Regression Analysis of Group Effects on Mediators

Model diagnostics indicated that the assumptions of linearity, homoscedasticity, and normality of residuals were met. In the models that adjusted for baseline R-GPTS score and each mediator (without covariate adjustment), most mediators, tested separately, did not differ significantly between intervention groups (Table 2). Belief inflexibility was the only mediator to show a statistically significant between-group difference, with a beta coefficient of 0.60 (95%CI: 0.02-1.18, *P* = .041; Table 2 and figure, Supplementary Data Content 6). This finding should be interpreted as indicating that changes in belief inflexibility are more strongly associated to changes in paranoia within the VR–CBTp group.

**Table 2:** Group Differences in Associations Between Changes in Mediators and Paranoia (Linear Regression Single-Mechanism Models Without Covariate Adjustment).

	Beta coefficient	SE	95%CI	*P*-value
SBQ positive	−0.01	0.08	−0.18 to 0.15	0.89
SBQ negative	0.02	0.16	−0.30 to 0.33	0.91
BCSS—negative others	−0.17	0.30	−0.75 to 0.42	0.58
BCSS—positive others	−0.34	0.33	−1.00 to 0.32	0.31
BCSS—negative self	0.09	0.29	−0.48 to 0.65	0.77
BCSS—positive self	−0.30	0.27	−0.83 to 0.23	0.26
DACOBS—belief inflexibility	0.60	0.29	0.02 to 1.18	0.041^*^

Beta coefficients from linear regression model analyses. All analyses are adjusted for R-GPTS total score and each mechanism measure at baseline. * indicates a *P*-value <.05. SBQ: Safety Behavior Questionnaire. BCSS: Brief Core Schema Scale. DACOBS: Davos Assessment of Cognitive Biases Scale.

In the models, also testing each mediator separately, which additionally controlled for age, biological sex assigned at birth, and depression (with covariate adjustment), no statistically significant between-group differences were observed for belief inflexibility or any other mediator (Table 3 and figure, Supplementary Data Content 6).

**Table 3:** Group Differences in Associations Between Changes in Mediators and Paranoia (Linear Regression Single-Mechanism Models with Covariate Adjustment).

	Beta coefficient	SE	95% CI	*P*-value
SBQ positive	−0.03	0.09	−0.20 to 0.14	0.75
SBQ negative	−0.001	0.16	−0.32 to 0.32	0.99
BCSS—negative others	−0.23	0.30	−0.82 to 0.36	0.44
BCSS—positive others	−0.32	0.34	−0.99 to 0.34	0.33
BCSS—negative self	−0.04	0.29	−0.62 to 0.54	0.89
BCSS—positive self	−0.29	0.27	−0.83 to 0.24	0.28
DACOBS—belief inflexibility	0.55	0.30	−0.03 to 1.13	0.06

Beta coefficients from linear regression model analyses. All analyses are adjusted for R-GPTS total score and each mechanism measure at baseline. All analyses are adjusted for the following covariates: age, biological sex assigned at birth, and depression defined as a score of ≥8 on the Calgary Depression Symptom Scale. SBQ: Safety Behavior Questionnaire. BCSS: Brief Core Schema Scale. DACOBS: Davos Assessment of Cognitive Biases Scale.

### Mediation Analysis Using SEM

The SEM analyses converged satisfactorily, yielding a stable solution. Each mediator was tested separately, and none showed statistically significant between-group differences in the indirect (mediating) effect (Table 4).

**Table 4:** Group Differences in Mediation Effects (SEM with Bootstrapping in Single-Mediator Models).

	Total effect Point estimate (bootstrap SE) (bootstrap CI lower to CI upper)	Direct effect Point estimate (bootstrap SE) (bootstrap CI lower to CI upper)	Indirect effect Point estimate (bootstrap SE) (bootstrap CI lower to CI upper)	Indirect/total
SBQ positive	−1.00 (1.71) (-4.29 to 2.42)	−0.50 (1.54) (-3.46 to 2.54)	−0.50 (0.76) (-2.00 to 1.08)	0.50
SBQ negative	−1.00 (1.71) (-4.35 to 2.48)	−1.30 (1.57) (-4.38 to 1.81)	0.30 (0.767 (-1.07 to 1.74)	−0.30
BCSS—negative others	−1.00 (1.71) (-4.40 to 2.52)	0.55 (1.53) (-2.44 to 3.71)	−1.55 (0.82) (-3.42 to 0.01)	1.55
BCSS—positive others	−1.00 (1.71) (-4.44 to 2.42)	−0.54 (1.65) (-3.92 to 2.79)	−0.46 (0.47) (-1.54 to 0.44)	0.46
BCSS—negative self	−1.00 (1.71) (-4.36 to 2.42)	−1.00 (1.64) (-4.21 to 2.30)	0.01 (0.47) (-1.02 to 0.90)	−0.01
BCSS—positive self	−1.00 (1.71) (-4.41 to 2.43)	−1.03 (1.69) (-4.46 to 2.27)	0.04 (0.26) (-0.53 to 0.69)	−0.04
DACOBS—belief inflexibility	−1.00 (1.71) (-4.38 to 2.37)	−1.51 (1.63) (-4.73 to 1.64)	0.51 (0.55) (-0.54 to 1.86)	−0.51

Values are presented as point est. (bootstrap SE) (bootstrap CI_lower – CI_upper) indicating point estimate of mediation effect, SE and 95% confidence intervals based on the percentile of the bootstrap distribution. Indirect/total indicates the proportion mediated. SBQ: Safety Behavior Questionnaire. BCSS: Brief Core Schema Scale. DACOBS: Davos Assessment of Cognitive Biases Scale.

### Post Hoc Analyses

For VR–CBTp, in-session exposure was significantly associated with paranoia reduction, both without (β = 0.05, 95% CI: 0.004-0.10, *P* = .034) and with (β = 0.05, 95% CI: 0.006-0.10, *P* = .028) covariate adjustment. Each additional minute of exposure was associated with a 0.05 increase in paranoia score at treatment cessation, indicating worse outcomes with greater amount of exposure. No such association was observed for standard CBTp (see table, Supplementary Data Content 7).

## Discussion

This is the first study to investigate a broad range of potential mechanisms of change in VR–CBTp and CBTp for SSD-related paranoia by directly comparing the two interventions. We hypothesized that changes in safety behaviors would play a stronger mediating role in VR–CBTp, whereas changes in self-related core beliefs and belief inflexibility would play a stronger mediating role in CBTp. These hypotheses were not supported. Across analyses examining each mediator separately, no evidence of differential mediation between interventions was observed. Correlational analyses indicated that changes in positive safety behaviors and negative self- and other-related core beliefs were associated with reductions in paranoia across both treatment conditions. Surprisingly, post hoc analyses examining the role of in-session exposure showed a marginally significant association in VR–CBTp, suggesting worse paranoia outcomes with greater in-session exposure. However, the effect was clinically negligible. No association was observed in standard CBTp.

Taken at face value, the findings suggest that the two interventions may be considered equivalent in terms of both efficacy^36^ and candidate mechanisms of change. Notably, a secondary moderation analysis,^37^ using data from the FaceYourFears trial, indicated that VR–CBTp may be more advantageous than standard CBTp among patients with high avolition and among those with moderate-to-high overall delusion severity, whereas standard CBTp may be more beneficial for patients with low overall delusion severity. Outside these subgroups, treatment selection may best be guided by factors such as patient preference, accessibility, and engagement.^12^^,^^36^

Compared with previous VR- and non-VR–based trials, our findings regarding safety behaviors diverge from those of the Pot-Kolder et al. 2018 VR–CBTp^14^ trial, which used the same software program and a similar treatment manual as ours, and the GameChange automated VR–CBTp^20^ trial, both of which reported mediation effects relative to inactive controls. Despite greater in-session exposure in our VR–CBTp group, safety behaviors were no more influential than in the CBTp group. Notably, the GameChange automated VR–CBTp^20^ trial did not assess core beliefs or belief inflexibility. Furthermore, in our trial, positive safety behaviors may have functioned as mediators to a similar extent across interventions.

The dose–response relationship between the amount of in-session exposure and paranoia reduction has not previously been examined, at least in VR–CBTp trials. A cautious interpretation of our findings could be that other therapeutic elements, when applied instead of exposure, may have contributed equally to treatment effects. If so, this may challenge the primacy of exposure proposed in theory^4^^,^^7^ and supported by earlier meta-analytic findings.^50^ Notably, the latter were based on treatment descriptions rather than actual treatment delivery. These findings warrant further investigation.

Importantly, our results on safety behaviors align with the THRIVE trial,^21^ which compared VR–CBTp with VR-based mental relaxation and found similar improvements in safety behaviors without differential mediation.

Findings regarding belief inflexibility vary across studies depending on measurement. The Garety et al. 2015 reasoning intervention trial,^24^ examined potential reasoning bias mediators, but not safety behaviors or core beliefs, and found mediation by Possibility of Being Mistaken (PM), although this effect disappeared after baseline adjustment. Both the SloMo^19^ trial, which also did not examine safety behaviors or core beliefs, and the FeelingSafe^18^ trial reported mediation consistent with PM. Notably, in the FeelingSafe trial^18^ only one participant received the “Reasoning bias module”, suggesting that PM was influenced when other mechanisms were addressed.

In the present study, we did not find any between-group difference for belief inflexibility as a mediator, and measurement differences may explain this discrepancy. The DACOBS belief inflexibility subscale^22^ used in our study was validated against dogmatism^51^ and assesses “delusion-neutral” rather than “delusion-specific” content, unlike PM.^23^ The Pot-Kolder et al., 2018 VR–CBTp^14^ and van den Berg et al. 2015 PTSD–psychosis^52^^,^^53^ trials, both employing the DACOBS belief inflexibility subscale, similarly failed to detect mediation. Collectively, this suggests that the DACOBS belief inflexibility subscale and PM may capture distinct constructs.

Regarding core beliefs, the Freeman et al., 2014 self-confidence^17^ and Phoenix^33^ trials suggested that positive self-related beliefs may function as mediators, and the FeelingSafe trial^18^ found that positive other-related beliefs mediated reductions in delusional conviction. In our study, findings differed somewhat, indicating a similar role for negative self- and other-related beliefs as mediators in both interventions. Within the FeelingSafe trial,^18^ negative self-related beliefs were hypothesized mediators of self-confidence but did not differ substantially by receipt of the self-confidence module, suggesting that they were affected when other mechanisms were targeted.

Taken together, findings from prior VR- and non-VR–based trials compared with inactive controls have at times been interpreted as indicative of intervention-specific mechanisms. However, selective inclusion of candidate mediators, differences in measurement approaches, and the lack of systematic cross-treatment comparisons have likely contributed to stronger inferences regarding intervention-specific mechanisms than the available evidence appears to support. Findings from both the THRIVE^21^ and FeelingSafe^18^ trials are also consistent with this more cautious interpretation. Specifically, neither the THRIVE^21^ trial nor our trial found evidence of differential mediation effects: for safety behaviors in THRIVE,^21^ and for safety behaviors, belief inflexibility, and core beliefs in our trial, relative to VR mental relaxation or standard CBTp with minimal exposure, respectively. This was despite the comparators being hypothesized to operate through substantially different mechanisms of change. Thus, when considered collectively, the available evidence does not justify interpreting findings within the field as indicative of intervention-specific mechanisms.

### Methodological Challenges

Alongside this, important methodological challenges regarding current mediation approaches in psychotherapeutic RCTs warrant attention. Our design relied on pre- and post-treatment assessments conducted outside therapy sessions. Most previous studies in the field have used comparable designs, with the THRIVE trial^21^ representing an exception through the inclusion of a mid-treatment assessment, also conducted outside therapy sessions.

This shared design limitation highlights a distinction that may be critical for mechanistic research on paranoia: that between intervention-specific *activating mechanisms* (processes directly engaged by treatment elements) and *downstream mechanisms* (broader changes emerging subsequently).^54^ Because assessments were limited to pre-, mid-, and post-treatment timepoints outside therapy sessions, we speculate that both our study and prior trials employing mediation analyses primarily captured non-specific downstream changes rather than processes proximal to specific treatment elements.^54^ If this is the case, the simple linear mediation models used in our study and prior trials are likely insufficient for modeling such downstream effects, as downstream mechanisms may represent end products of complex, non-linear processes.^54^ It is also possible that the identified mediators reflect non-mechanistic constructs associated with general improvement rather than causal processes.^55^ Accordingly, mediation models embedded within traditional RCT designs of psychotherapeutic interventions may be inherently limited in their ability to examine intervention-specific activating mechanisms.

A related methodological challenge concerns the multicomponent nature of psychotherapeutic interventions in traditional RCTs. Interventions are delivered as “treatment packages” comprising multiple elements, making it inherently difficult to isolate the effects of individual elements.^54^^,^^55^ In our study, exposure was hypothesized to be the key therapeutic element in VR–CBTp, but this was not supported, exemplifying the limitations of delivering treatment as “packages”, in which elements assumed to be most important may not, in fact, be so. Mediation analyses from the OPTIMIZE trial,^56^ a 2^4^ factorial optimization RCT targeting social anxiety, further highlight these challenges, showing that individual treatment elements may influence multiple mediators, while single mediators may be affected by several elements. In that study, psychoeducation and exposure emerged as particularly influential treatment elements compared with cognitive restructuring and attention training, influencing both safety behaviors and negative social cognitions.

Within this context, two alternative explanations for our findings merit consideration. First, treatment elements and mediators may relate to one another in many-to-many ways, potentially violating key assumptions underlying the applied mediation model in ways not adequately accommodated by the analyses.

Second, the interventions may not have been sufficiently distinct. Both were grounded in cognitive models, delivered by the same therapists with a risk of treatment contamination, and likely shared a similar emphasis on psychoeducation, which may have constituted an important unmeasured mechanism of change.

To address these methodological challenges, future research should investigate mechanisms with greater temporal precision, for example through ecological momentary assessment,^57^ and place greater emphasis on in-session processes to better capture activating mechanisms.^13^^,^^54^ Alternative study designs beyond formal mediation analyses may also prove valuable. For example, Freeman et al.’s 2016 VR study^13^ showed greater improvement when participants were encouraged to drop safety behaviors than when encouraged to behave as usual within the same VR environment, thereby providing experimental support for the causal role of reducing safety behaviors. Finally, future studies should aim to isolate and examine specific treatment elements.

### Future Directions

At the same time, we caution against increasing the rigidity of the treatment manuals in psychotherapeutic interventions solely to demonstrate specific mechanisms of change. Excessive rigidity may compromise clinical responsiveness^58^ and the therapeutic alliance.^59^ From a mechanistic perspective, however, a causal interventionist framework supports isolating candidate processes and testing whether their manipulation leads to change in paranoia.^4^^,^^58^ This may be achieved without introducing excessive rigidity by employing inclusion criteria and outcome measures grounded in specific mechanisms and their hypothesized effects on paranoia. Exclusion criteria should, in turn, be kept to a minimum to ensure generalizability. Once identified, such mechanisms can inform mechanism-targeted treatment elements that are incorporated into flexible, modular programs, thereby allowing treatment to be tailored to individuals’ primary complaints.^18^ In this way, mechanistic precision and clinical flexibility are complementary rather than competing aims. Through modular design and continued identification of targetable processes, preferably through co-development with people with lived experience,^60^^,^^61^ psychotherapeutic treatment of paranoia may better align with patient preferences.^62^ This may strengthen the therapeutic alliance^59^ and ultimately improve treatment efficacy.

Additionally, there is a need to standardize key therapeutic measures to enhance comparability across studies, and an international consensus on core measures in paranoia, with regular updates, may now be timely.

### Strengths and Limitations

A strength of the study is the cautious interpretation of marginally significant findings, given the risk of false positives without multiplicity correction. In addition, the analyses were based on data from a high-quality RCT.

Our study has important limitations. First, the absence of an inactive control group means we cannot exclude that within-group changes reflect factors such as natural illness course, service engagement, or reporting bias rather than intervention-specific effects. Nonetheless, there is evidence to suggest that genuine treatment effects are present. The Pot-Kolder et al. 2018 VR–CBTp trial^14^ reported moderate to large between-group effects on GPTS subscales when comparing a similar VR–CBTp intervention with a TAU waitlist condition, in which participants received TAU while awaiting treatment.^14^ Moreover, in our trial, R-GPTS scores in both groups shifted from levels typical of psychotic clinical groups at baseline to those characteristic of non-psychotic clinical groups (anxiety or depression) at post-treatment and 6-month follow-up.^43^

Second, additional mechanisms identified in cognitive models, such as worry and sleep disturbances,^4^^,^^18^ as well as mechanisms related to metacognitive strategies, such as overconfidence to errors bias,^27^ may have contributed but were not assessed.

Third, concurrent assessment precludes establishing temporal order; thus, the results can examine associations but not causality.^63^

Finally, selection bias cannot be excluded in the present PP sample with complete data. However, no between-group differences were found in the proportion of completers and non-completers. Furthermore, the ITT sample and the PP sample with complete data did not differ in baseline characteristics or in analyses examining changes from baseline to treatment cessation.^36^ Taken together, this suggests a low risk of selection bias and ITT mediation analyses would be unlikely to provide additional insight.

## Conclusion

In conclusion, this is the first study to examine a range of potential mechanisms of change in VR–CBTp and CBTp for SSD-related paranoia. None of the tested mediators, thought to represent theorized mechanisms, differed between the interventions. These findings suggest that the therapeutic effects of CBTp and VR–CBTp may not rely on distinct mechanisms and treatment selection may instead be guided by factors such as symptom profile, patient preference, accessibility, and engagement. However, advancing mechanism research in paranoia may require greater conceptual and temporal precision, for instance by distinguishing activating mechanisms from downstream effects and isolating specific treatment elements. Designs capable of tracking and isolating such processes over time, as well as well-designed alternative study approaches, will be essential for generating more definitive mechanistic insights.

## Supplementary Material

Supplementary_material_FaceYourFears_SBO_correct_attribution_sgag028

## Data Availability

Details about the FaceYourFears trial are publicly available on ClinicalTrials.gov (identifier: NCT04902066). The complete study protocol and its amendment have been published in *Trials*. All de-identified data from the trial are stored permanently at the Danish National Archives (Rigsarkivet), which serves as a public repository. Requests for data access can be submitted through https://www.rigsarkivet.dk. Access is governed by Danish legislation, including the Archives Act (Arkivloven), the Archives Executive Order (Arkivbekendtgørelsen), the General Data Protection Regulation (Databeskyttelsesforordningen), and the Danish Data Protection Act (Databeskyttelsesloven), due to the classification of these data as sensitive personal information. During the first 20 years, access requires prior evaluation by the research team and must always be approved by the Danish Data Protection Agency (Datatilsynet). After 75 years, the data will be freely accessible without restrictions. The research team supports data sharing with academic or clinical researchers engaged in non-commercial, ethically approved projects. An initial response to any request will be provided within one month, and a Data Access Agreement must be completed before data are released.

## Study Approval Statement

The trial was approved by the Capital Region of Denmark Committee on Health Research Ethics (H-20048806) and the Danish Data Protection Agency (P-2020-823).

## Code Availability

The code is available at https://codeberg.org/VIRTU/faceyourfears.
