# Supplementary material for: Potential Mechanisms of Change in Virtual Reality-based Cognitive Behavioural Therapy and Standard Cognitive Behavioural Therapy for Schizophrenia-Related Paranoia: A Mediation Analysis from the FaceYourFears Trial
Source: Schizophr Bull Open. 2026 Jul 21;7(1):sgag028. doi: 10.1093/schizbullopen/sgag028 (PMC13485156; doi:10.1093/schizbullopen/sgag028)
Supplement: Supplementary file 1 [file supplementary_material_faceyourfears_sbo_correct_attribution_sgag028.docx]

# Supplementary Data Content 1 | Description of the experimental (VR-CBTp) and comparator (standard CBTp) interventions

*The experimental intervention,* VR-CBTp, integrates core CBTp techniques with a VR-enhanced behavioural component primarily targeting safety behaviours. The intervention involves identifying safety behaviours and helping participants recognise these as maladaptive coping strategies that may provide short-term relief but maintain distress in the long term. Through behavioural exercises in VR, participants are supported in gradually reducing these behaviours (e.g. making eye contact with others). This process aims to promote more adaptive appraisals of others’ intentions and facilitate the disconfirmation of maladaptive beliefs, thereby reducing paranoia.

The structured intervention comprises initial psychoeducation, individualised case formulation, and progressive VR exposure (a few minutes in sessions 1–2, approximately 15 minutes in session 3, and 15–30 minutes in sessions 4–9), with session 10 dedicated to evaluation and planning for independent practice. Participants are further encouraged to engage in real-life exposure between sessions based on situations practised in VR.

The VR intervention is delivered through an animated three-dimensional programme comprising five everyday social environments: a bus, café, park, shopping street, and supermarket. These environments are designed to simulate situations in which paranoia is likely to arise. Participants are immersed both auditorily and visually and can navigate the environments using a joystick, interact with virtual characters, engage in role-play exercises, test threat beliefs, and explore worst-case scenarios. Simulations can be repeated to support the rehearsal of new strategies.

The therapist, physically present in the same room, communicates with the participant via a headset while monitoring and controlling the virtual environment. During role-play, the therapist can speak through virtual characters using a modified voice and switch back to their own voice to provide guidance. The intervention is tailored to the individual, with difficulty adjusted across sessions by modifying key features such as the number and behaviour of virtual characters, social interactions, eye contact, and background noise. These adjustments are made dynamically in response to the participant’s progress to maintain an appropriate level of challenge. Participants can leave the virtual environment at any time, either by removing the headset or by requesting a pause, with immediate support from the therapist.

Together, this flexible and individualised approach aims to maintain therapeutic effectiveness while minimising the risk of excessive distress.

*The comparator*, standard CBTp, primarily focuses on cognitive restructuring and metacognitive strategies with a particular focus on reasoning bias such as belief inflexibility. Behavioural interventions using in-vivo exposure are conducted in sessions when feasible.

Session 1 involves case formulation and treatment planning. Subsequent sessions provide psychoeducation on belief inflexibility and other maintaining factors of paranoia, alongside analyses of triggering situations to critically evaluate automatic thoughts. For instance, *cumulative probability calculations* can be used to directly target belief inflexibility by counteracting premature inferences and introducing the possibility of being mistaken. Session nine is specifically dedicated to addressing self-esteem and maladaptive self-related core beliefs and session ten reviews the therapy course and support participants in planning future psychotherapeutic work after treatment. Homework is planned and scheduled to reinforce between-session learning between each session and is tailored to session content.

# Supplementary Data Content 2 | Clinical and sociodemographic characteristics of the intention-to-treat sample at baseline

| Variable | VR-CBTp | CBTp | Total |
| --- | --- | --- | --- |
|  |  |  |  |
|  |  |  |  |
| Age in years | 27.7 (23.0-35.0)^a^, n=126 | 26.5 (22.7-31.8)^a^ ,n=128 | 26.8 (22.8-33.1)^a^, n=254 |
| Sex |  |  |  |
| Female | 72(57.1%) | 74 (57.8%) | 146 (57.5%) |
| Male | 54 (42.9%) | 54 (42.2%) | 108 (42.5%) |
|  |  |  |  |
| Education |  |  |  |
| Primary (in Denmark up until age 14) | 38 (30.2%) | 44 (34.4%) | 82 (32.3%) |
| Secondary | 43 (34.1%) | 51 (39.8%) | 94 (37.0%) |
| Vocational education | 17 (13.5%) | 12 (9.4%) | 29 (11.4%) |
| University degree | 25 (19.9%) | 18 (14.1%) | 43 (16.9%) |
| Other^b^ | 3 (2.4%) | 3 (2.3%) | 6 (2.4%) |
|  |  |  |  |
| Occupational status |  |  |  |
| Employment (full-time or part-time) | 17 (13.5%) | 27 (21.1%) | 44 (17.3%) |
| Student | 19 (15.1%) | 28 (21.9%) | 47 (18.5%) |
| Unemployment | 58 (46.0%) | 52 (40.6%) | 110 (43.3%) |
| Disability retirement (or retired) | 25 (19.8%) | 17 (13.3%) | 42 (16.5%) |
| Housewife or husband | 1 (0.8%) | 0 | 1 (0.4%) |

# Supplementary Data Content 2 (continued) | Clinical and sociodemographic characteristics of the intention-to-treat population at baseline

| Variable | VR-CBTp | CBTp | Total |
| --- | --- | --- | --- |
|  |  |  |  |
| Diagnosis |  |  |  |
| F20 Schizophrenia | 94 (74.6%) | 90 (70.3%) | 184 (72.4%) |
| F21 Schizotypal disorder | 20 (15.9%) | 27 (21.1%) | 47 (18.5%) |
| F22 Paranoid psychoses | 6 (4.7%) | 4 (3.1%) | 10 (3.9%) |
| F25 Schizoaffective psychosis | 5 (4.0%) | 3 (2.3%) | 8 (3.2%) |
| F29 Non-organic psychosis | 1 (0.8%) | 4 (3.1%) | 5 (2.0%) |
|  |  |  |  |
| Outpatient routine care setting |  |  |  |
| Early intervention services (OPUS) | 59 (46.8%) | 63 (49.2%) | 122 (48.0%) |
| Flexible Assertive Community teams (F-ACT | 67 (53.2%) | 62 (48.4%) | 129 (50.8%) |
| Other | 0 | 3 (2.3%) | 3 (1.2%) |
|  |  |  |  |
| Olanzapine equivalent (mg/day) | 10.1 (3.4-20)^a^, n=126 | 10.1 (5-18.2)^a^, n=128 | 10.1 (0-18.9)^a^, n=254 |

# Supplementary Data Content 2 (continued) | Clinical and sociodemographic characteristics of the intention-to-treat population at baseline

| Variable | VR-CBTp | CBTp | Total |
| --- | --- | --- | --- |
|  |  |  |  |
| Participation in additional group psychoeducation as part of TAU from baseline to treatment cessation | 14 (11.1%) | 10 (7.8%) | 24 (9.5%) |
| Participation in additional group social skills training as part of TAU from baseline to treatment cessation | 11 (8.7%) | 14 (10.9%) | 25 (9.8%) |
| Participation in additional individual psychological counseling as part of TAU from baseline to treatment cessation | 5 (4.0%) | 6 (4.7%) | 11 (4.3%) |
| Participation in additional other psychosocial intervention^c^ as part of TAU from baseline to treatment cessation | 21 (16.7%) | 13 (10.2%) | 34 (13.4%) |
|  |  |  |  |
| R-GPTS total | 28.3 (25.9-30.8%), n=126 | 29.3 (26.6-31.9%), n=128 |  |
| SAPS composite total | 22.9 (20.6-25.0%), n=126 | 21.8 (19.7-23.9%), n=128 |  |
|  |  |  |  |
| In-session exposure in minutes | 83.5 (74.7-92.3%), n=126 | 8.0 (4.3-11.6%), n=128 |  |

Selected results from: Jeppesen, U.N., Vernal, D.L., Due, A.S. *et al.* Virtual reality-based versus standard cognitive behavioral therapy for paranoia in schizophrenia spectrum disorders: a randomized controlled trial. *Nat Med* **31**, 3425–3439 (2025). https://doi.org/10.1038/s41591-025-03880-8. Licensed under CC BY 4.0 (https://creativecommons.org/licenses/by/4.0/). Values are presented as n(n%), indicating the frequency and its

# Supplementary Data Content 2 (continued) | Clinical and sociodemographic characteristics of the intention-to-treat population at baseline

corresponding percentage of the total and n(n-n%), (n) indicating the mean value and its corresponding 95 % confidence and its standard deviation. n= indicates number of observations. ^a^ :indicates the median and its 25^th^ and 75^th^ percentile, presented as n(n-n), in non-normal distributed samples. ^b^ :Education: 'Other' refers to the lack of completion of Primary Education. ^c^ : ”other psychosocial intervention” refers primarily to family intervention. TAU: Treatment as usual. R-GPTS Revised Green Paranoid Thought Scale. SAPS: Scale for the Assessment of Positive Symptoms.

# Supplementary Data Content 3 | Between-group adjusted mean differences and effect size estimates for the R-GPTS and the potential mediators: results from the main intention-to-treat analysis of the primary study

| Variable | VR-CBTp mean (95%CI) | CBTp mean (95%CI) | Adjusted mean difference (standard error) | 95%CI for the adjusted mean difference;  p value | Cohen’s d |
| --- | --- | --- | --- | --- | --- |
| R-GPTS total |  |  |  |  |  |
| Baseline | 28.3 (25.9-30.8%)  n=126 | 29.3 (26.6-31.9%)  n=128 |  |  |  |
| Treatment cessation | 17.0 (15.1-18.8%) | 17.6 (15.6-19.6%) | 0.65 (1.38) | (-2.06 to 3.37%); 0.64 | 0.05 |
| SBQ total (negative + positive) |  |  |  |  |  |
| Baseline | 55.1 (51.0-59.2%)  n=126 | 54.2 (50.0-58.4%)  n=128 |  |  |  |
| Treatment cessation | 36.3 (33.7-39.0%) | 38.6 (35.8-41.5%) | 2.28 (1.97) | (-1.62 to 6.18%)  ; 0.25 | 0.15 |
| SBQ negative |  |  |  |  |  |
| Baseline | 38.9 (37.1-40.8%)  n=126 | 38.3 (36.4-40.2%)  n=128 |  |  |  |
| Treatment cessation | 9.3 (8.1-10.4%) | 9.3 (8.0-10.6%) | 1.06^a^ (0.30) | (0.59 to 1.90%)^a^  ; 0.85 | 0.02^b^ |
| DACOBS - Belief inflexibility |  |  |  |  |  |
| Baseline | 21.0 (20.1-22.0%)  n=126 | 21.2 (20.3-22.2%)  n=128 |  |  |  |
| Treatment cessation | 20.5 (19.7-21.2%) | 19.9 (19.1-20.7%) | -0.55 (0.56) | (-1.66 to 0.56%); 0.33 | -0.13 |

# Supplementary Data Content 3 (continued) | Between-group adjusted mean differences and estimated effect sizes for R-GPTS and the potential mediators: Results from the main intention-to-treat analysis

| BCSS – negative self |  |  |  |  |  |
| --- | --- | --- | --- | --- | --- |
| Baseline | 10.1 (9.2-11.0%)  n=126 | 10.2 (9.4-11.1%)  n=128 |  |  |  |
| Treatment cessation | 7.8 (7.1-8.4%) | 8.6 (7.9-9.4%) | 0.87 (0.50) | (-0.12 to 1.87%); 0.09 | 0.20 |
| BCSS – negative others |  |  |  |  |  |
| Baseline | 7.5 (6.7-8.4%)  n=126 | 8.7 (7.8-9.6%)  n=128 |  |  |  |
| Treatment cessation | 5.7 (5.0-6.4%) | 6.6 (5.9-7.4%) | 1.16^a^ (0.23) | (0.73 to 1.84%)^a^; 0.52 | 0.081^b^ |
| BCSS – positive self |  |  |  |  |  |
| Baseline | 8.1 (7.1-9.0%)  n=126 | 7.5 (6.7-8.3%)  n=128 |  |  |  |
| Treatment cessation | 8.8 (0.4%) | 8.7 (8.0-9.5%) | -0.05 (0.51) | (-1.05 to 0.95%); 0.92 | -0.02 |
| BCSS – positive others |  |  |  |  |  |
| Baseline | 9.1 (8.4-9.9%)  n=126 | 8.5 (7.8-9.1%)  n=128 |  |  |  |
| Treatment cessation | 10.2 (9.5-10.9%) | 9.8 (9.1-10.6%) | -0.34 (0.52) | (-1.38 to 0.67%); 0.50 | -0.08 |

Selected results from: Jeppesen, U.N., Vernal, D.L., Due, A.S. *et al.* Virtual reality-based versus standard cognitive behavioral therapy for paranoia in schizophrenia spectrum disorders: a randomized controlled trial. *Nat Med* **31**, 3425–3439 (2025). https://doi.org/10.1038/s41591-025-03880-8. Licensed under CC BY 4.0 (https://creativecommons.org/licenses/by/4.0/). Between-group adjusted mean difference after adjusting for biological sex assigned at birth, study site and dichotomised symptom severity of GPTS subscale *Ideas of Persecution* (≥45 or <45 at baseline), along with the estimated effect size. All analyses are linear regression models based on the intention-to-treat principle and handled with multiple imputations. All analyses are adjusted for baseline measurement of each outcome. All analyses are conducted without adjustment for baseline imbalances. Values are presented as n(n-n%), indicating the mean value and its 95 % confidence interval, n= indicates number of observations. ^a^ indicates that a log transformation was applied, and the reported result is therefore an exponentiated, back-transformed value. ^b^ indicates that the Cohen’s d has been calculated by using the log-transformed mean and standard deviation values for the two groups. Time points: Treatment cessation, mean = 4.5 months (95%CI 4.3-4.6) after baseline. R-GPTS: Green Paranoid Thought Scale. SBQ: Safety Behaviour Questionnaire. DACOBS: DACOBS: Davos Assessment of Cognitive Biases Scale. BCSS: Brief Core Schema Scale.

# Supplementary Data Content 4 | Between-group adjusted mean differences for the R-GPTS and the potential mediators: results from the per-protocol sensitivity analysis of the primary study

| R-GPTS total |  |  |  |  |
| --- | --- | --- | --- | --- |
| Treatment cessation | 16.4 (14.4-18.3%) | 17.5 (15.5-19.5%) | 1.12 (1.41) | (-1.67 to 3.90%); 0.43 |
| SBQ total (negative + positive |  |  |  |  |
| Treatment cessation | 36.5 (33.8-39.3%) | 39.0 (36.1-41.9%) | 2.48 (2.01) | (-1.51 to 6.48%)  ; 0.22 |
| SBQ negative |  |  |  |  |
| Treatment cessation | 9.1 (7.8-10.3%) | 9.4 (8.1-10.7%) | 1.13^a^ (0.30) | (0.63 to 2.02%)^a^  ; 0.69 |
| DACOBS - Belief inflexibility |  |  |  |  |
| Treatment cessation | 20.5 (19.7-21.3%) | 19.9 (19.1-20.7%) | -0.65 (0.58) | (-1.80 to 0.51%); 0.27 |
| BCSS – negative self |  |  |  |  |
| Treatment cessation | 7.7 (7.1-8.3%) | 8.2 (7.5-8.9%) | 0.51 (0.47) | (-0.42 to 1.44%); 0.28 |
| BCSS – negative others |  |  |  |  |
| Treatment cessation | 5.5 (4.8-6.3%) | 6.4 (5.6-7.2%) | 1.08^a^ (0.25) | (0.66 to 1.77%)^a^; 0.76 |
| BCSS – positive self |  |  |  |  |
| Treatment cessation | 8.8 (8.0-9.5%) | 8.8 (8.1-9.6%) | 0.06 (0.53) | (-0.98 to 1.11%); 0.90 |
| BCSS – positive others |  |  |  |  |
| Treatment cessation | 10.2 (9.5-11.0%) | 9.9 (9.1-10.7%) | -0.33 (0.54) | (-1.40 to 0.75%); 0.55 |

Selected results from: Jeppesen, U.N., Vernal, D.L., Due, A.S. *et al.* Virtual reality-based versus standard cognitive behavioral therapy for paranoia in schizophrenia spectrum disorders: a randomized controlled trial. *Nat Med* **31**, 3425–3439 (2025). https://doi.org/10.1038/s41591-025-03880-8. Licensed under CC BY 4.0 (https://creativecommons.org/licenses/by/4.0/). Only participants receiving full treatment (10 sessions) are analysed (VR-CBTp: n=102, CBTp: n=97). Between-group adjusted mean difference after adjusting for biological sex assigned at birth, study site and dichotomised

# Supplementary Data Content 4 | Between-group adjusted mean differences for the R-GPTS and the potential mediators: results from the per-protocol sensitivity analysis of the primary study

symptom severity of GPTS subscale *Ideas of Persecution* (≥45 or <45 at baseline). All analyses are conducted without adjustment for baseline imbalances. All analyses are linear regression models handled with multiple imputations. All analyses are adjusted for baseline measurement of each outcome. Values are presented as n(n-n%), indicating the mean value and its 95 % confidence interval. ^a^ indicates that a log transformation was applied, and the reported result is therefore an exponentiated, back-transformed value. ^b^ indicates a p value < 0.05. Time points only for participants receiving full treatment: Treatment cessation, mean = 4.5 months (95%CI 4.3-4.7) after baseline; Follow-up, mean = 10.5 months (95%CI 10.2-10.7) after baseline. Baseline values and Cohen’s d are not reported in the sensitivity analyses in the primary study. R-GPTS: Green Paranoid Thought Scale. SBQ: Safety Behaviour Questionnaire. DACOBS: DACOBS: Davos Assessment of Cognitive Biases Scale. BCSS: Brief Core Schema Scale.

# Supplementary Data Content 5 | Pearson correlations between the R-GPTS total score and potential mediators at baseline, treatment cessation and change from baseline to treatment cessation

|  | Safety behaviours | |  | Core beliefs | | | |
| --- | --- | --- | --- | --- | --- | --- | --- |
|  | Positive | Negative | Belief inflexibility | Positive self | Negative self | Positive others | Negative others |
| Baseline | 0.21** | 0.17* | 0.24*** | -0.12 | 0.21** | -0.33*** | 0.40*** |
| Treatment cessation | 0.42*** | 0.35*** | 0.28*** | -0.16* | 0.28*** | -0.27*** | 0.47*** |
| Change between timepoints^a^ | 0.19** | 0.17* | 0.14 | -0.02 | 0.19** | -0.17* | 0.21** |

^a^Change between timepoints reflects the correlation between changes in R-GPTS and the individual mediator from baseline to treatment cessation, serving as a crude indicator of potential mediation across groups. * indicates p-value < 0.05, ** indicates p-value <0.01 and *** indicates p-value < 0.001.

# Supplementary Data Content 6 | Visualisation of linear regression model analyses of group effects on belief inflexibility

The model without covariate adjustment is adjusted for baseline paranoia and belief inflexibility, and the model adjusted for covariates is further adjusted for age, biological sex assigned at birth, and baseline depression (binary). “assignment 1” is CBTp standard and “assignment 2” is VR-CBTp.

Alternative text: Two-panel plot illustrating the relationship between belief inflexibility and paranoia severity (R-GPTS total score) in the CBTp and VR-CBTp groups. The left panel shows the model without covariate adjustment, adjusted only for baseline paranoia and baseline belief inflexibility, whereas the right panel shows the model with covariate adjustment, additionally adjusted for age, sex assigned at birth, and baseline depression. In both models, higher belief inflexibility is associated with higher paranoia scores, with a steeper positive association observed in the VR-CBTp group than in the CBTp group. The interaction term is statistically significant in the model without covariate adjustment (p = 0.041) but not in the model with covariate adjustment (p = 0.064).

# Supplementary Data Content 7 | Association between amount of in-session exposure and paranoia reduction in each group with and without covariate adjustment.

|  | Beta coefficient | SE | 95% CI | p-value |
| --- | --- | --- | --- | --- |
| VR-CBTp without covariates | 0.05 | 0.02 | 0.004 to 0.10 | 0.034* |
| VR-CBTp with covariates | 0.05 | 0.02 | 0.006 to 0.10 | 0.028* |
| Standard CBTp without covariates | -0.01 | 0.05 | -0.11 to 0.08 | 0.80 |
| Standard CBTp with covariates | 0.01 | 0.05 | -0.10 to 0.09 | 0.89 |

Beta coefficients from linear regression model analyses. All analyses are adjusted for R-GPTS total score at baseline. “With covariates”-analyses are adjusted for the following covariates: age, biological sex assigned at birth, and depression defined as a score of ≥8 on the Calgary Depression Symptom Scale. * indicates a p-value <0.05.
